# A deep learning approach to predict temporal changes of subdural hemorrhage on computed tomography

**DOI:** 10.1038/s41598-025-21721-z

**Published:** 2025-10-29

**Authors:** M. S. F. Fasla, D. M. T. K. Dissanayake, D. M. I. Dhananjaya, Mohan L. Jayatilake, P. B. Hewavithana

**Affiliations:** 1 Department of Radiography /Radiotherapy, Faculty of Allied HealthSciences, University of Peradeniya, Peradeniya, 20400 Sri Lanka; 2 Department of Radiology, Faculty of Medicine, University of Peradeniya, Peradeniya, 20400 Sri Lanka

**Keywords:** Subdural hemorrhage, Computed tomography, Convolutional neural network, Computational biology and bioinformatics, Medical research, Neurology

## Abstract

Subdural hemorrhage (SDH) is a critical condition requiring prompt assessment of its progression using computed tomography (CT). This study aimed to develop a deep-learning model to predict temporal changes in SDH by leveraging Hounsfield Units (HU) to estimate hemorrhage age across acute, subacute, and chronic stages. A total of 825 pre-processed CT slices from the RSNA dataset were balanced across SDH stages and analyzed using a convolutional neural network (CNN) implemented in Python on Google Colab. Model performance was evaluated using accuracy, sensitivity, specificity, precision, F1-score, and the Area Under the Receiver Operating Characteristic Curve (AUC-ROC). The model achieved 83.11% training accuracy and 85.33% prediction accuracy. Sensitivity for acute, subacute, and chronic SDH was 86.67%, 84%, and 85.33%, respectively, with specificity values of 94%, 88%, and 96%. Precision scores were 87.84%, 77.78%, and 91.43%, while F1 scores were 87.25%, 80.77%, and 88.28%. AUC-ROC values ranged from 0.9394 to 0.9731 across five folds, reflecting robust classification performance. The results highlight the model’s potential to support radiologists as a second-reader tool, streamline emergency triage, and enhance diagnostic efficiency within clinical workflows.

**Supplementary Information:**

The online version contains supplementary material available at 10.1038/s41598-025-21721-z.

## Introduction

### Subdural hemorrhage

Subdural hemorrhage (SDH) is the most common type of intracranial hemorrhage (ICH), characterized by the accumulation of blood in the subdural space^1^. It can occur in any age group^1^, primarily due to blunt head injury^2^. SDH commonly results from stretching and tearing of the bridging cortical veins as they traverse the subdural space to drain into an adjacent dural sinus^2,3^. Sudden velocity changes in the head create shearing forces that rupture these veins^1,3,4^. In some cases, the arachnoid may also be torn, resulting in a mixture of blood and cerebrospinal fluid (CSF) in the subdural space^2,5^. SDH typically presents as a crescent-shaped collection between the dural and arachnoid layers, which is larger and more diffuse than extradural hematomas^6^. Unlike extradural hemorrhage, SDH is not limited by sutures but rather by dural reflections such as the falx cerebri, tentorium, and falx cerebelli^2,7,12^.

### Pathophysiology of SDH formation

The mechanism of SDH formation involves sudden changes in velocity, causing shearing forces, which lead to vein rupture^8^. Acute SDH presents as a crescent-shaped area of hyperdensity on non-contrast computed tomography (NCCT) images, extending diffusely over the cerebral hemisphere. The clot retraction process increases density to typically > 50–60 Hounsfield Units (HU). Up to 40% of cases exhibit mixed hyper or hypodensity due to unclotted blood, serum, or CSF from arachnoid laceration^9^. Rarely, acute SDH may appear nearly isodense in patients with coagulopathies or severe anemia due to reduced hemoglobin concentration and the absence of clot formation^9–11^. Subacute SDH, typically occurring within 3–21 days, gradually becomes isodense to the adjacent cortex as the density decreases to ~ 35–40 HU due to aging blood clots and protein degradation^11^. Chronic SDH, which is at least 3 weeks old, becomes hypodense relative to the cortex and may resemble CSF or a subdural hygroma. In approximately 10–30% of chronic SDH cases, recurrent bleeding occurs due to stretched cortical veins or vascularized neomembranes, forming a hematocrit level commonly observed posteriorly^2,12^.

### Diagnostic modalities for SDH

SDH is primarily diagnosed using computed tomography (CT) and magnetic resonance imaging (MRI). CT is preferred due to its widespread availability, rapid acquisition, short scan time, and high sensitivity to blood^12,13^. Brain window images are commonly acquired with a 5 mm slice thickness. Contrast-enhanced CT (CECT) or MRI can be used to overcome challenges in identifying isodense or bilateral subdural collections, especially in the subacute phase^2,14^.

### Imaging characteristics of SDH

CT imaging reveals different characteristics of SDH depending on the phase of hemorrhage. Acute SDH is typically seen as hyperdense, crescent-shaped collections with a density greater than 50 HU^2,13,15^. Subacute SDH, which occurs between 3 and 21 days, becomes isodense to the adjacent cortex^16^. Chronic SDH, which presents as hypodense collections with a density reaching approximately 0 HU, is isodense to CSF. In some cases, chronic SDH may evolve into a biconvex shape or show signs of acute rebleeding^17^.

### Challenges in SDH diagnosis

In some instances, hyperacute SDH appears isodense due to a swirled appearance from a mixture of clot, serum, and ongoing unclotted blood^2,18^. Acute-on-chronic SDH presents additional complexity, often characterized by hypodensity with hematocrit levels in patients with clotting disorders or those on anticoagulants^2,19^. Identifying SDH in the subacute phase can be particularly challenging due to its isodense nature, requiring indirect signs such as sulcal effacement and midline shift for diagnosis^2,20^.

### Advances in artificial intelligence for SDH detection

Artificial intelligence (AI), particularly machine learning and deep learning, has transformed medical imaging. Machine learning uses algorithms to analyze large datasets, enabling automated disease detection and treatment planning^21–25^. Deep learning, a subset of machine learning, employs convolutional neural networks (CNNs) to automate tasks such as detecting ICH in CT scans, extracting features, and optimizing model performance. This enhances diagnostic accuracy, supports decision-making, and aids in prognosis^26–29^.

#### Literature survey

The detection and analysis of ICH, particularly SDH, in CT brain images have seen significant advancements through various imaging techniques and machine learning models. Kyeong-Seok Lee et al. focused on evaluating the density changes in SDH over time using CT scans. Their study revealed that acute SDH is typically hyperdense, transitioning to hypodense within 3 weeks, with chronic SDH showing increased density up to 90 days before decreasing again, a phenomenon attributed to microhemorrhages and neomembrane maturation^30^.

**Fig. 1:**
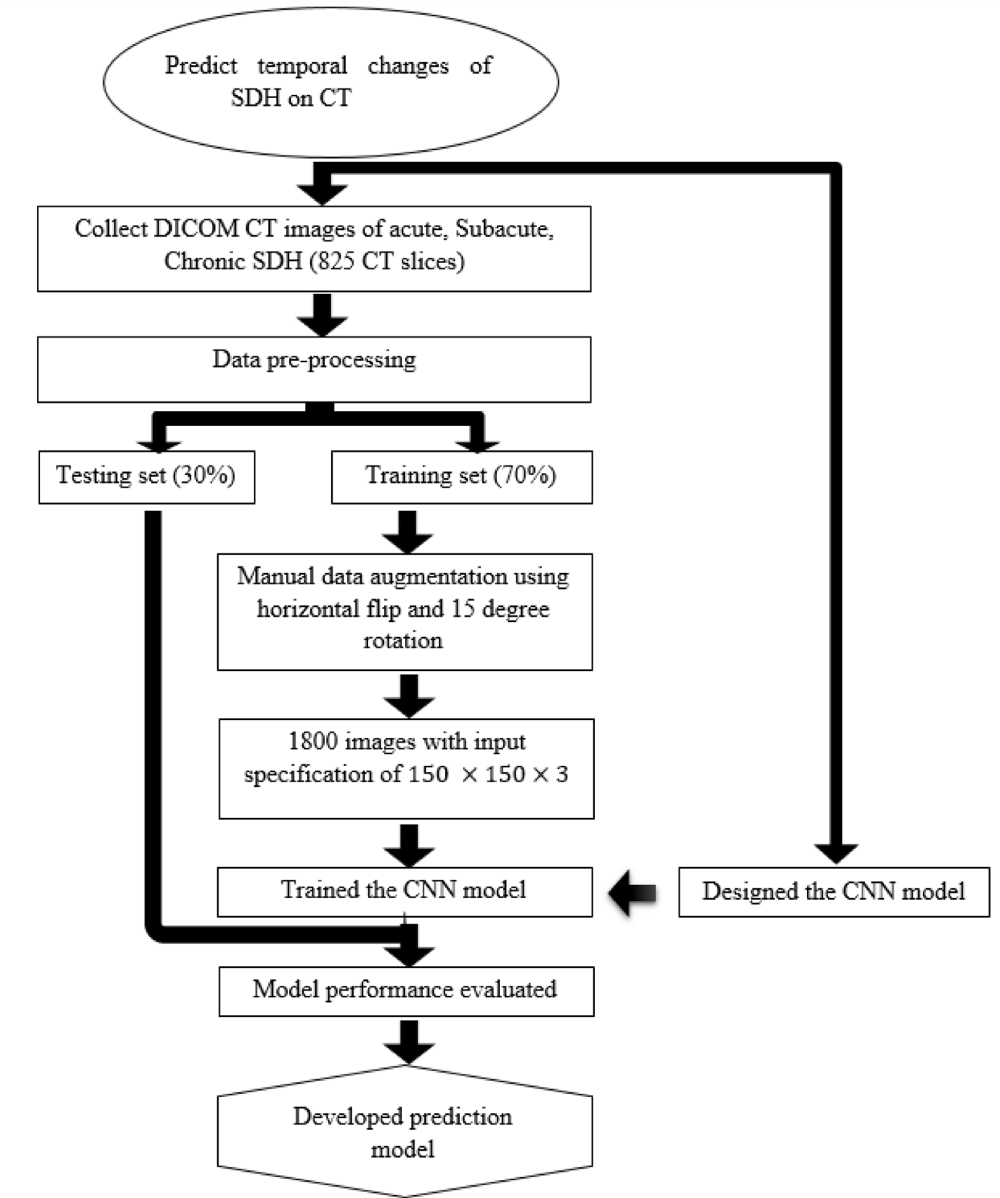
The workflow for predicting SDH on CT images using a convolutional neural network (CNN) is outlined. The methodology comprises data collection, preprocessing, augmentation, model development, and evaluation.

In recent years, machine learning has played a pivotal role in automating hemorrhage detection. Muntakim Mahmud Khan et al. developed a deep learning-based approach using U-Net and other architectures for image segmentation, achieving notable success in detecting ICH with a DSC of 85.76%^31^. Similarly, Maya and Asha proposed an automated framework for hemorrhage classification, utilizing U-Net for segmentation and CapsNet for classification, reaching a training accuracy of 97.3% and a validation accuracy of 92.1%^32^. In another study, Shelke et al. developed an intelligent system for acute brain hemorrhage diagnosis, using spatial fuzzy c-means clustering and active contours for image segmentation, achieving a precision of 1.0 and recall of 0.9583^33^. Dr. Sofia Bobby and Vishal evaluated various segmentation techniques, with neural network-based segmentation achieving the highest accuracy of 98.70%^34^. Additionally, Praveen Kumaravel et al. explored different deep learning frameworks, including a modified AlexNet-SVM classifier, which reached an impressive accuracy of 99.86% in classifying CT images into hemorrhage and non-hemorrhage categories^35^. These studies collectively highlight the potential of deep learning not only to detect but also to predict the evolution of SDH in CT scans, offering a foundation for future research into automating the prediction of temporal changes in SDH (Fig. 1).

## Justification

There have been significant advancements in the automated detection of ICH and its differentiation into subtypes. However, most previous studies, such as those by Muntakim Mahmud Khan et al. (2023), Maya B.S. and Asha T. (2020), and others, focused primarily on detecting ICH and classifying hemorrhages at specific time points, without addressing the evolving nature of SDH over time. These studies successfully applied machine learning and deep learning techniques to detect and classify various types of hemorrhages, but they did not address the prediction of temporal changes in SDH, which is a critical aspect of its management. This gap was particularly important, as detecting the time-dependent progression of SDH on CT images remains a challenge, often requiring expert radiologists to interpret subtle changes. SDH is a critical medical condition that demands rapid diagnosis and intervention. Timely identification of its progression is crucial for effective treatment and management. Despite its clinical importance, existing methods do not adequately address the need for automated systems capable of predicting the temporal evolution of SDH on CT scans. The present study was conducted to fill this gap by developing a deep learning model, specifically a CNN, to predict the temporal changes of SDH using CT images. CNNs have demonstrated remarkable success in medical image analysis due to their ability to learn spatial hierarchies and detect complex patterns in imaging data^26–29^. By leveraging this model, the study enhanced diagnostic accuracy and improved the ability to track SDH progression over time. The potential impact of this research is global, as it could contribute to the development of a novel, highly accurate tool for real-time monitoring and prediction of SDH, ultimately improving patient outcomes worldwide. This study developed a robust deep learning algorithm to detect and classify SDH by creating a predictive model leveraged HU data to estimate the temporal progression and age of SDH on CT.

## Results

Table 1 illustrates the CNN model’s performance across various metrics. The training accuracy of the model, which reflects how well the model fits the training data, was 83.11 $\:\pm\:$ 2.74%, indicating that it accurately captured relevant features during training. On the test set, the model achieved a prediction accuracy of 85.33%, demonstrating its ability to correctly classify images across the different SDH categories.

**Fig. 2:**
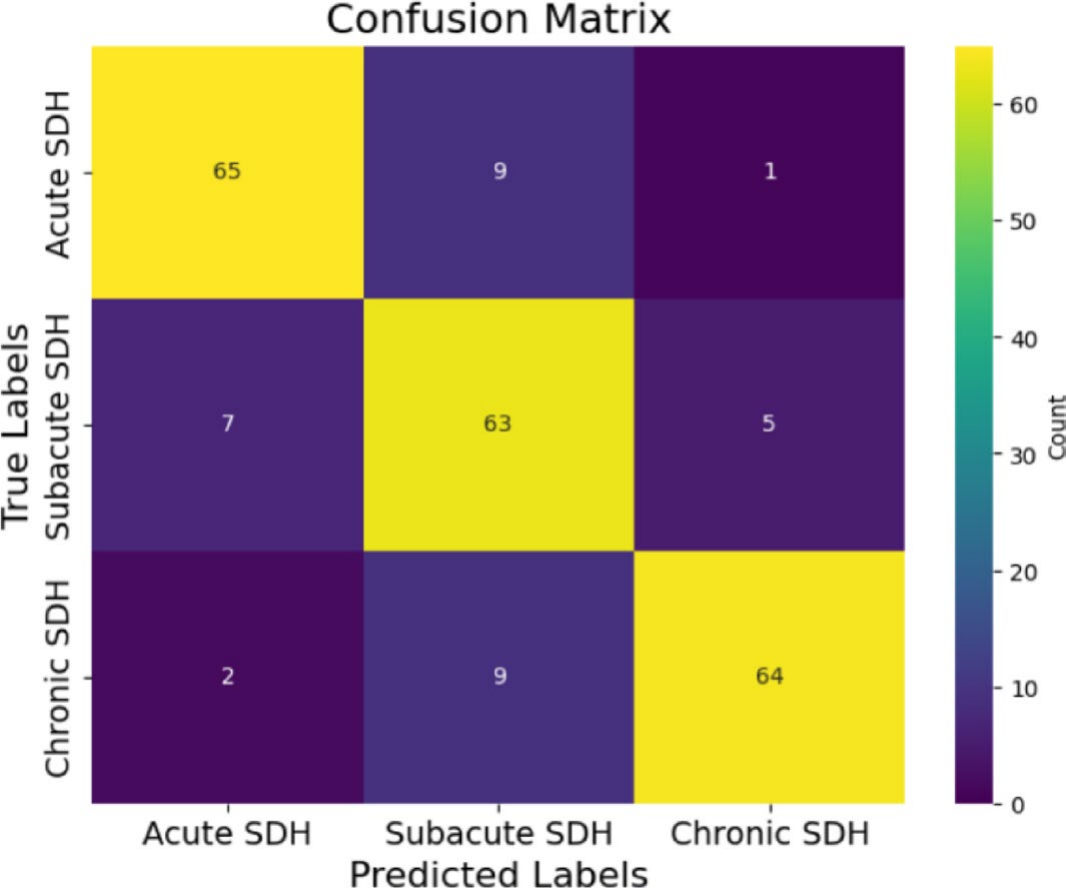
Confusion matrix of the proposed model (3 × 3). According to the confusion matrix, the model predicted 65 out of 75 cases of acute SDH, 63 out of 75 cases of subacute SDH, and 64 out of 75 cases of chronic SDH.

The model’s ability to correctly identify SDH cases was assessed through sensitivity (recall). Sensitivity was 86.67% for acute SDH, 84% for subacute SDH, and 85.33% for chronic SDH, indicating that the model effectively predicted most SDH categories while minimizing false negatives. Conversely, the specificity of the model, measuring its ability to correctly classify non-hemorrhage cases, was 94% for acute SDH, 88% for subacute SDH, and 96% for chronic SDH, indicating its capacity to reduce false positives (Fig. 2).

The precision of the model, which quantifies the proportion of true positives among all predicted positives, was 87.84% for acute SDH, 77.78% for subacute SDH, and 91.43% for chronic SDH. This suggests that the model’s positive classifications were largely accurate. The F1-score, which balances both precision and recall, was 87.25% for acute SDH, 80.77% for subacute SDH, and 88.28% for chronic SDH, providing a single metric to assess the model’s performance in terms of both false positives and false negatives.

For classification performance, we additionally calculated the Dice Similarity coefficient (DSC) and Intersection over Union (IoU) from the confusion matrix at the case level. The model achieved DSC scores of 87.25% for acute SDH, 80.77% for subacute SDH, and 88.28% for chronic SDH. The corresponding IoU scores were 77.38%, 67.74%, and 79.01% for acute, subacute, and chronic SDH, respectively. Although these metrics are conventionally used in segmentation, here they were adapted for classification to provide an additional measure of overlap between predicted and true labels. For completeness, we also adapted IoU and DSC from confusion matrices as exploratory overlap metrics; however, because these measures are conventionally applied in segmentation, their detailed results are provided in the Supplementary Material (see Table S1). Conventional mask-based IoU/DSC will be reported in future segmentation work.

**Fig. 3:**
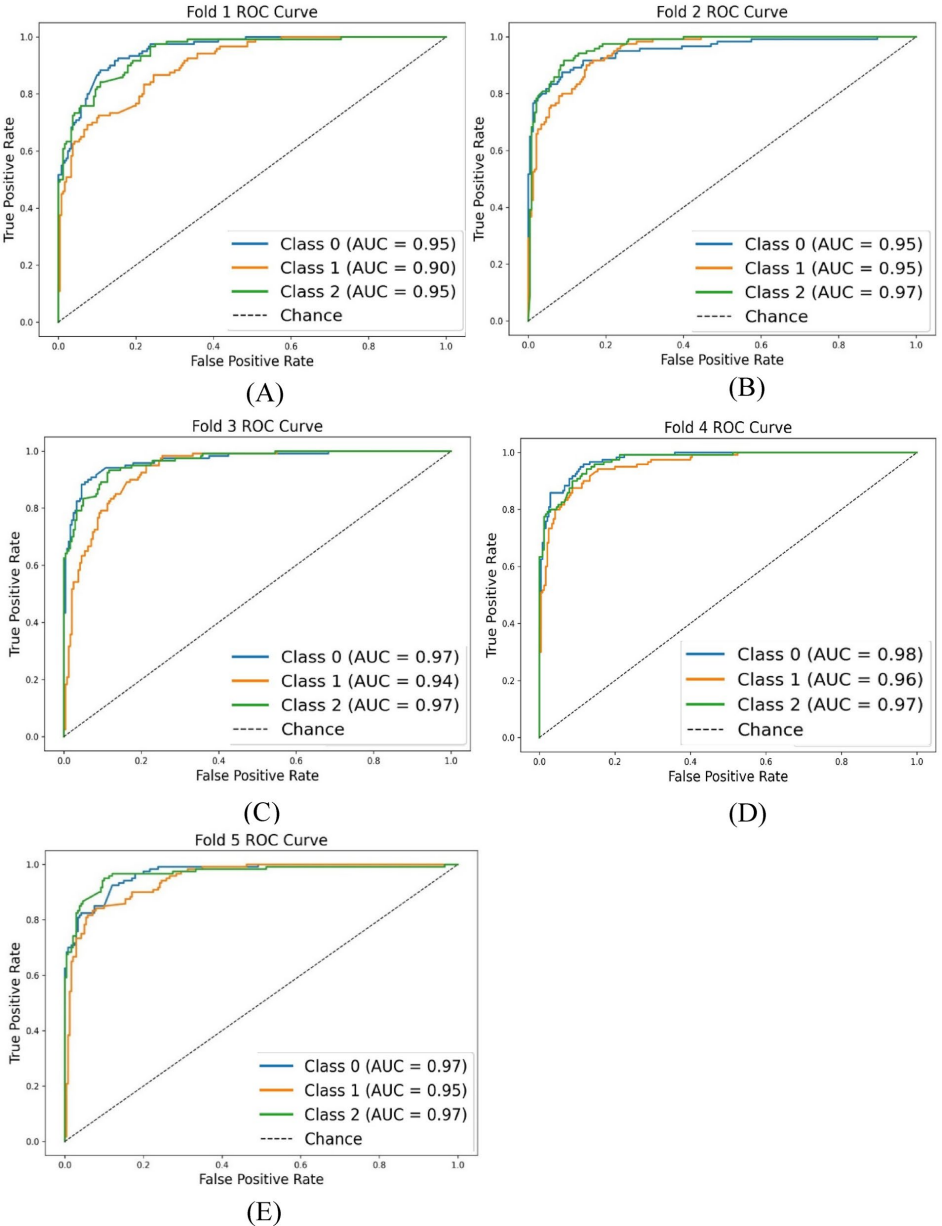
AUC value and ROC curve for each k-fold, where Class 0 refers to acute SDH, Class 1 refers to subacute SDH, and Class 2 refers to chronic SDH (**A**) to (**E**) represent AUC values and ROC curves for the 1st to 5th k-folds.

The Area Under the Receiver Operating Characteristic Curve (AUC-ROC) scores (see Fig. 3) ranged from 0.9394 to 0.9731 across the five folds, indicating that the model’s ability to distinguish between positive and negative SDH cases was robust and reliable throughout the cross-validation process. An AUC-ROC value close to 1 further suggests that the model exhibited excellent discriminatory power in each fold, effectively identifying both positive and negative cases.

To assess the level of agreement between the model’s predictions and the radiologist-provided ground truth, Cohen’s kappa was calculated. This metric was used to quantify expert–model agreement, as inter-rater agreement among multiple radiologists was not assessed in this study. The model achieved a Cohen’s kappa of 0.78 on the internal test set, indicating substantial concordance between the model’s predictions and the expert annotations. This further supports the model’s consistency and potential clinical utility.

Overall, the proposed model demonstrated strong performance across all evaluation metrics, achieving high training accuracy and effective classification. The model’s high sensitivity, specificity, precision, and excellent AUC-ROC values, along with substantial agreement as reflected by Cohen’s kappa, underscore its potential as a reliable tool for the prediction of temporal changes in SDH.

**Table 1:** Performance matrics of the CNN model for SDH classification and segmentation.

Metric		Acute SDH	Subacute SDH	Chronic SDH	Overall
Training accuracy		–	−	−	83.11 $\:\pm\:$ 2.7408%
Prediction accuracy		−	−	−	85.33%
Accuracy		91.56%	86.67%	92.44%	−
Sensitivity		86.67%	84.00%	85.33%	−
Specificity		94.00%	88.00%	96.00%	−
Precision		87.84%	77.78%	91.43%	−
F1-score		87.25%	80.77%	88.28%	−
AUC	K-fold 1	0.95	0.90	0.95	0.9394
	K-fold 2	0.95	0.95	0.97	0.9596
	K-fold 3	0.97	0.94	0.97	0.9556
	K-fold 4	0.98	0.96	0.97	0.9731
	K-fold 5	0.97	0.95	0.97	0.9642
Cohen’s kappa (K)		–	–	–	0.78

**Fig. 4:**
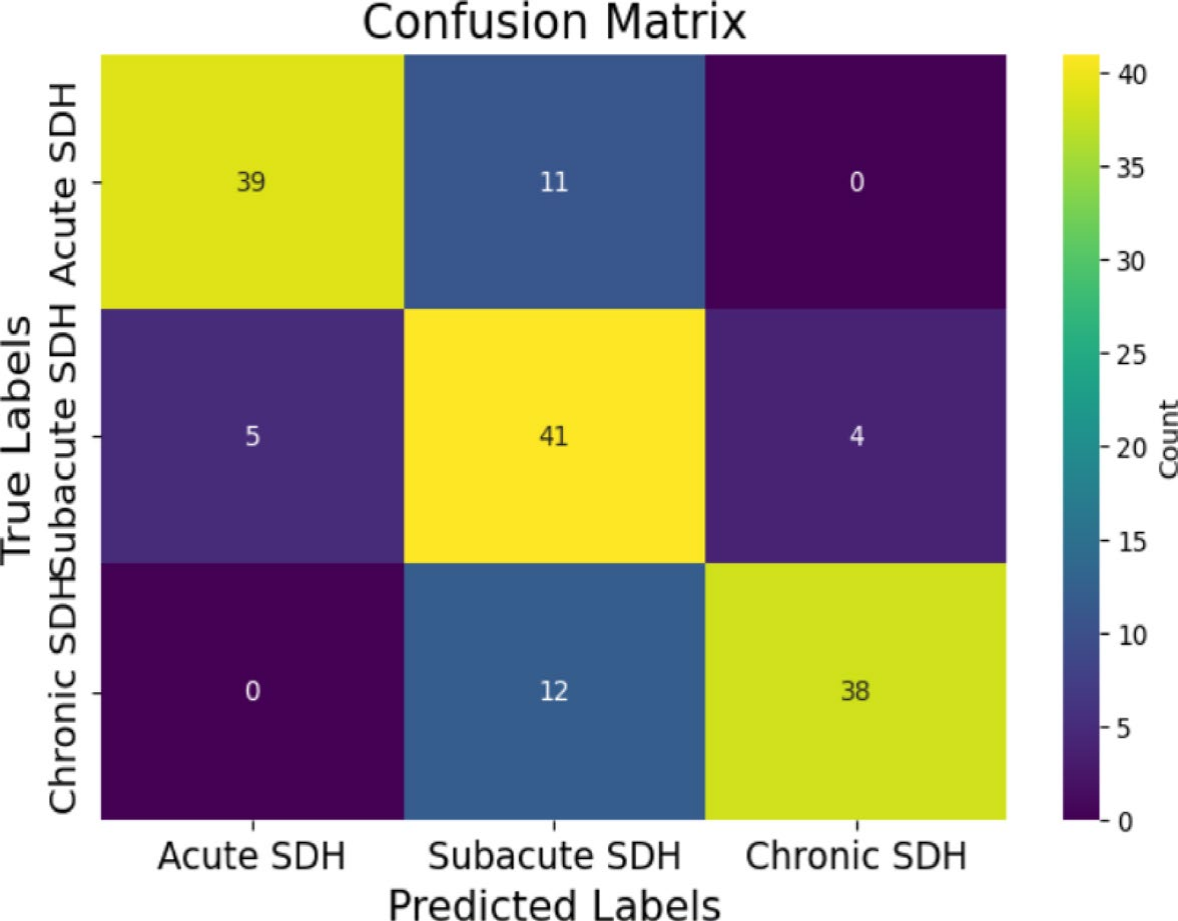
The external validation confusion matrix of the proposed model (3 × 3). According to the confusion matrix, the model predicted 39 out of 50 cases of acute SDH, 41 out of 50 cases of subacute SDH, and 38 out of 50 cases of chronic SDH.

To ensure the generalizability and reliability of the developed algorithms and models across diverse patient populations and imaging protocols, independent validation was conducted using a distinct dataset. Specifically, 50 separate slices from each classification category, acute, subacute, and chronic SDH, were extracted from the RSNA dataset for evaluation purposes. Model performance was assessed using a comprehensive set of metrics, including accuracy, specificity, sensitivity, precision, F1-score, and mean absolute error (MAE). A 3 × 3 confusion matrix (Fig. 4) was employed to facilitate a detailed performance analysis. The external validation results indicated strong predictive capabilities across all SDH categories. As detailed in Table 2, the model achieved classification accuracies of 89.33% for both acute and chronic SDH, and 78.67% for subacute SDH. Sensitivity scores were 78% for acute, 82% for subacute, and 76% for chronic cases, reflecting the model’s competence in identifying true positives. High specificity values of 95% for acute, 77% for subacute, and 96% for chronic SDH highlighted the model’s ability to correctly classify negative cases. Precision rates were similarly robust, with 88.64% for acute, 64.06% for subacute, and 90.48% for chronic SDH, indicating a strong correspondence between predicted and actual positive cases. The MAE values further supported these findings, with minimal error observed: 0.1067 for both acute and chronic, and 0.2133 for subacute SDH. Additionally, Cohen’s kappa(K) for this external dataset was 0.68, indicating moderate agreement between the model’s predictions and expert annotations, thereby supporting the model’s reliability and real-world applicability. The modest reduction in K compared to the internal validation value of 0.78 likely reflects greater heterogeneity in acquisition protocols and case mix, which our model did not explicitly normalize. Collectively, these results affirm the model’s effectiveness, consistency, and applicability across varied clinical scenarios.

**Table 2:** External validation results of SDH stages, evaluating accuracy, sensitivity, specificity, precision, F1-score, MAE, and Cohen’s kappa.

Metric	Acute SDH	Subacute SDH	Chronic SDH	Overall
Accuracy	89.33%	78.67%	89.33%	−
Sensitivity	78.00%	82.00%	76.00%	−
Specificity	95.00%	77.00%	96.00%	−
Precision	88.64%	64.06%	90.48%	−
F1-score	82.98%	71.93%	82.61%	−
MAE	0.1067	0.2133	0.1067	−
Cohen’s kappa (K)	−	−	−	0.68

## Discussion

This study presents a novel application of deep learning, specifically CNNs, to classify the stages of SDH- acute, subacute, and chronic- while predicting their temporal progression based on CT imaging. Unlike most prior research, which primarily focused on detecting acute SDH or general hemorrhagic conditions, this work uniquely addresses the dynamic evolution of SDH over time, filling a significant gap in the literature^31–38^.

One of the most significant contributions of this study is its novel approach to predicting the temporal changes of SDH, an area largely unexplored in prior research^30–38^. While Kyeong-Seok Lee et al. emphasized the importance of temporal dynamics in SDH, their work relied on manual longitudinal analysis rather than leveraging AI^30^. Our model’s capability to predict SDH progression using static CT images offers a groundbreaking tool for clinicians, especially in scenarios where serial imaging is not feasible.

Similarly, Muntakim Mahmud Khan et al. used deep learning to detect ICH, achieving an accuracy of 85.75%. However, their work focused primarily on detecting hemorrhages without distinguishing between different SDH stages or addressing the temporal evolution of SDH^31^. Our model expands on this by accurately classifying not only the presence of hemorrhage but also its stage (acute, subacute, or chronic) and its progression over time. Additionally, studies such as those by Maya and Asha employed CNN-based approaches for hemorrhage classification but did not explore temporal prediction, which is critical for clinical interventions^32^. Our model introduces this novel dimension, enhancing clinical utility.

Our model achieved an accuracy of 85.33% across the three SDH categories. This performance is comparable to, or even exceeds, previously reported accuracies for classification tasks involving hemorrhagic conditions using CT imaging^37^. For instance, earlier studies have successfully identified acute SDH but often neglected the subacute and chronic stages, or relied on manual feature extraction, which limited scalability^33,38^. In contrast, our CNN-based approach utilizes automated feature extraction, enabling more precise and efficient classification across all stages of SDH.

Smith et al. reported an accuracy of ~ 87% in detecting acute SDH using deep learning techniques. However, their model did not address the classification of subacute and chronic SDH or incorporate temporal progression, which are critical for clinical decision-making^37^. Compared to such efforts, our model offers a broader scope and greater clinical utility by enabling stage-specific classification and time-sensitive predictions.

Despite these promising results, our study is not without limitations. The dataset comprised 825 CT slices, which may not sufficiently capture the diversity of imaging conditions, patient demographics, or clinical scenarios. Larger datasets, reflecting varied clinical contexts, are necessary to enhance the model’s robustness and generalizability. Additionally, variations in image acquisition protocols, such as differences in CT scanners and reconstruction algorithms, were not fully accounted for, which could impact the model’s performance in real-world settings.

In order to ensure consistent and reliable labeling, cases presenting clinical ambiguity, such as mixed-density subdural hematomas, scans with poor image quality, or those with overlapping Hounsfield Unit characteristics, were intentionally excluded during dataset curation. Recognizing their importance for real-world applicability, future studies will aim to incorporate such diagnostically complex cases to enhance the model’s robustness and clinical relevance.

Another challenge lies in reducing false positives and false negatives, which can have serious clinical implications. While the accuracy achieved is noteworthy, future refinements should focus on minimizing misclassifications through improved model architecture, ensemble learning methods, and rigorous external validation. Prior studies have similarly reported challenges with misclassification in deep learning models for medical imaging, underscoring the importance of continuous model optimization^31–38^.

Integrating additional data sources, such as serial CT scans, clinical history, or biomarkers, represents an exciting avenue for future research. These enhancements could improve the model’s ability to predict not only the stage but also the trajectory of SDH, providing clinicians with actionable insights for personalized treatment planning.

## Conclusion

This study demonstrates the potential of deep learning, particularly CNNs, in classifying SDH stages- acute, subacute, and chronic- and predicting their temporal progression (Supplementary Figure S1) based on CT imaging. The model achieved an impressive classification accuracy of 85.33%, underscoring its ability to discern age-dependent variations in hemorrhage characteristics.

Our findings highlight the value of AI-driven tools in automating and enhancing diagnostic processes, offering clinicians a reliable method for assessing and monitoring SDH progression over time. By accurately identifying SDH stages and predicting their evolution, this approach can improve clinical decision-making, potentially reducing diagnostic time and enhancing patient outcomes.

In practical clinical settings, the model can be integrated into existing workflows via Picture Archiving and Communication System (PACS), enabling automated SDH classification directly within routine radiology infrastructure. It may serve as a second-reader tool to support radiologists, reduce interpretation time, and improve diagnostic consistency. Furthermore, the model could serve as a triage aid in emergency departments, helping prioritize acute cases and alleviate workload in high-pressure environments.

This research represents a significant step toward the integration of deep learning techniques into routine clinical workflows, paving the way for more efficient and accurate management of traumatic brain injuries. With continued advancements, AI-based models have the potential to revolutionize the diagnosis and treatment of SDH, ultimately improving care for patients worldwide.

## Methods

### Dataset acquisition

This study employed a retrospective quantitative approach. Ethical clearance was obtained from the Ethics Review Committee of the Faculty of Allied Health Sciences, University of Peradeniya, ensuring the privacy and confidentiality of all subjects. The dataset, made available by Kaggle, was fully anonymized prior to release and did not require individual informed consent. As the analysis involved only secondary use of this anonymized public dataset, the requirement for informed consent was waived by the Ethics Review Committee. CT images were sourced from the Radiological Society of North America (RSNA) dataset (https://www.kaggle.com/competitions/rsna-intracranial-hemorrhage-detection/data) and all data usage complied with the terms and conditions set by Kaggle. The RSNA dataset is a multi-institutional and multinational collection of brain hemorrhage CT images, and is the largest public dataset of its kind, including expert annotations from a large cohort of 15 volunteer neuro-radiologists for classifying ICH. For this study, only SDH images were selected. A total of 825 CT images were used, evenly distributed across three categories: acute, subacute, and chronic SDH (275 images per category). Inclusion criteria comprised male and female patients with CT brain scans demonstrating SDH. CT images from different types of CT machines were included in the study. Exclusion criteria eliminated images with artifacts, mixed hemorrhage, coexisting brain abnormalities, and low-quality scans. The best slices demonstrating hemorrhage were selected in the subdural/brain window (WL: 50–100 HU, WW: 130–300 HU) and categorized by HU ranges: acute (≥ 55 HU), subacute (25–50 HU), and chronic (10–20 HU), under the supervision of an experienced board-certified radiologist. HU ranges served as reference values to guide interpretation, but final labeling was based on radiologist judgment, integrating density and morphological features. Although a comparison with an HU rule-based classifier was considered, it was not included due to the retrospective nature of ground-truth labeling without ROI-based segmentation. For descriptive reporting, HU values were retrospectively measured by placing manual ROIs within the visually homogeneous hematoma region, using DICOM rescale slope/intercept for HU conversion. Each CT slice was treated as an independent sample. The dataset was split into training (70%) and testing (30%) subsets. The workflow of the prediction model is depicted in Fig. 1.

### Preprocessing

**Fig. 5:**
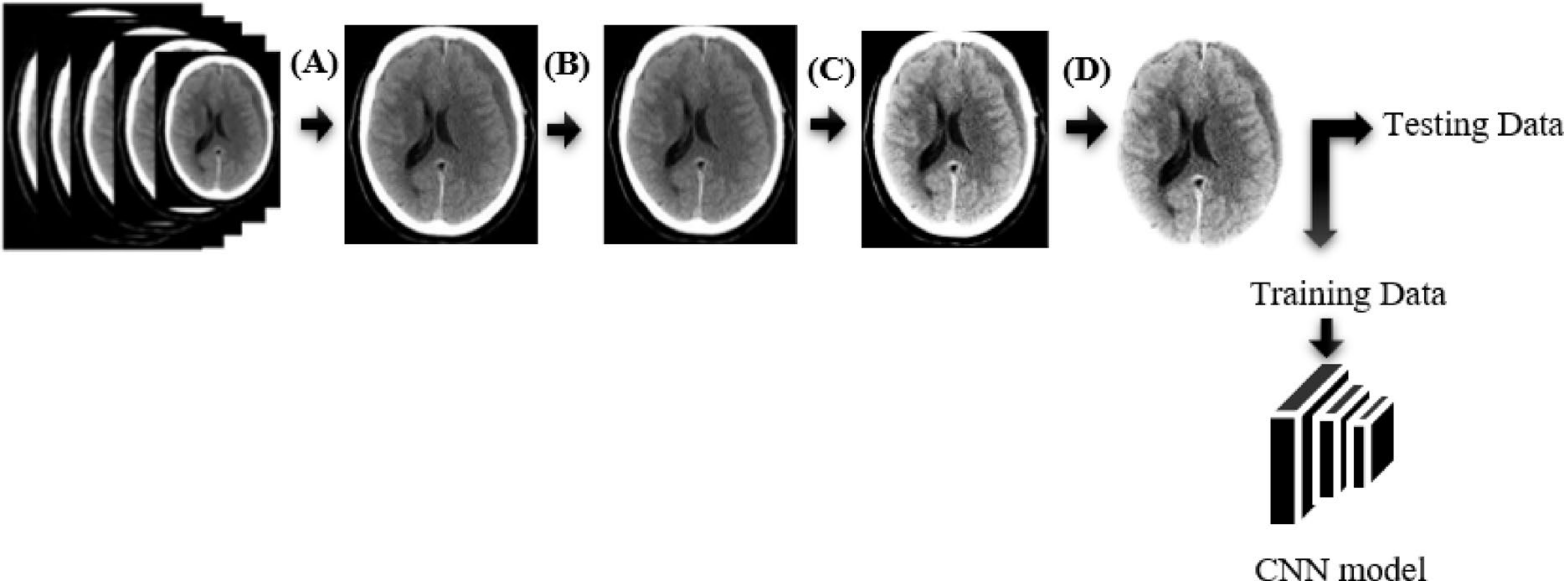
Pre-processing pipeline applied to the medical image data before being fed into a CNN model, with the steps of image conversion from DICOM to JPG (**A**), normalization (**B**), smoothing using a Gaussian filter 5 × 5 (**C**), and skull stripping (**D**). Window width (WW) and window level (WL) were kept constant across all panels. The processed data were then split into training and testing datasets for the CNN.

Data preprocessing was conducted using Google Colab with Python version 3.7 to enhance image quality and reduce noise. As shown in Fig. 5, raw CT DICOM images were converted to JPG format, with labeling and ROI visualization performed using a brain window (WL: 50–100 HU, WW: 130–300 HU). Preprocessing retained the full dynamic range and then linearly rescaled the images to 8-bit (0–255) for model input without applying lossy compression. This was followed by masking and normalization. Gaussian smoothing with a 5 × 5 kernel was applied to reduce noise and improve clarity. Skull stripping was performed to remove extraneous cranial structures, optimizing the dataset for analysis. This step required fine-tuning the threshold values for accurate segmentation.

### Data augmentation

Data augmentation was performed on the training set to improve model generalization. Two geometric transformations were applied: horizontal flipping and rotation by 15 degrees. These transformations expanded the training dataset from 600 to 1,800 images. All images were resized to dimensions of 150 × 150 × 3 (RGB channels) for input into the CNN model.

### Convolutional neural network architecture

**Fig. 6:**
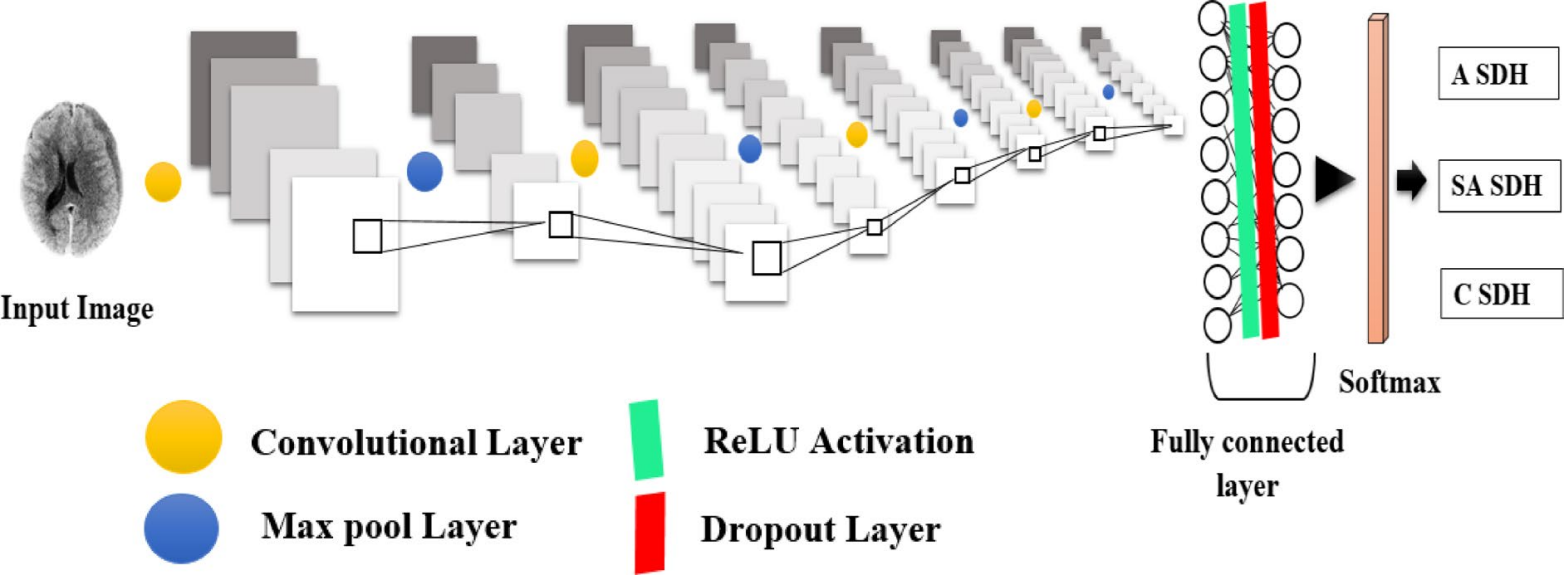
The proposed CNN architecture of the model consists of four convolutional layers (yellow dot), each followed by ReLU activation and max-pooling layers (blue dot). Convolution filters of size 3 × 3 and max-pooling with 2 × 2 windows were applied. The fully connected layers contained 512 units with 50% dropout (red line) to mitigate overfitting, and a softmax output layer was utilized to classify images into acute, subacute, and chronic SDH categories, denoted as ‘ASDH,’ ‘SASDH,’ and ‘CSDH,’ respectively.

A CNN was developed to classify SDH into three stages: acute, subacute, and chronic. The model architecture, shown in Fig. 6, was designed to extract relevant spatial and intensity-based features from brain CT images while maintaining computational efficiency. Input images were preprocessed and resized to 150 × 150 × 3, balancing sufficient resolution for hemorrhage detection with memory and processing efficiency on GPU/TPU hardware.

The network comprises four convolutional layers with 3 × 3 kernels and ReLU activation, each followed by a 2 × 2 max-pooling layer. This setup allows hierarchical feature extraction: early layers detect low-level features such as edges and textures, while deeper layers capture complex patterns differentiating SDH stages. The use of 3 × 3 filters is based on their proven effectiveness in capturing fine-grained local patterns, while max pooling reduces spatial dimensions and introduces translational invariance, making the network robust to variations in hemorrhage location.

The convolutional stack is followed by a fully connected dense layer with 512 units and ReLU activation, which integrates high-level features. A dropout layer with a 50% rate is used to reduce overfitting by preventing co-adaptation of neurons. The final output layer uses a softmax activation function to enable multiclass classification across the three SDH categories. The model was implemented in Python version 3.7 and trained on Google Colab with GPU acceleration.

For optimization, the model employed the Adam optimizer with a fixed learning rate of 1e–4, a choice guided by Adam’s ability to adaptively adjust learning rates for each parameter, leading to faster and more stable convergence on medical image data. The model was trained for 30 epochs with a batch size of 32. No learning rate decay or scheduling was applied, as empirical testing showed that the fixed learning rate yielded consistent convergence without oscillations. To evaluate model performance and prevent overfitting, a five-fold stratified cross-validation was performed.

### Model evaluation

The model’s performance was evaluated using several metrics derived from the confusion matrix (see Fig. 2), which categorizes predictions into four groups. True Positives (TP) represent SDH images that were correctly classified by the model. True Negatives (TN) are defined perclass in a one-vs-rest (OvR) scheme, representing samples from other SDH categories that were correctly identified as not belonging to the target class. False Positives (FP) refer to images from other categories that were incorrectly classified as belonging to the target class, while False Negatives (FN) represent images from the target class that were mistakenly identified as belonging to another category. Several important performance metrics are derived from this confusion matrix.

### Accuracy

The proportion of all correct predictions (both TP and TN) out of the total number of predictions. It indicates how often the model is correct

1 $$\:Accuracy=\frac{TP+TN}{TP+FP+TN+FN}\times\:100$$

### Sensitivity (recall)

Also known as the TP rate, this metric measures the proportion of actual positive cases (SDH) that are correctly identified by the model. It indicates the model’s ability to correctly identify positive cases.

2 $$\:Sensitivity\:\left(Recall\right)=\frac{TP}{TP+FN}\times\:100$$

### Specificity

Also known as the TN rate, this metric measures the proportion of actual negative cases (non-SDH) that are correctly identified by the model. It indicates the model’s ability to correctly identify negative cases.

3 $$\:Specificity=\frac{TN}{TN+FP}\times\:100$$

### Precision

Also known as the Positive Predictive Value (PPV), this metric measures the proportion of positive predictions that are actually correct. It indicates how accurate the model is when predicting the positive class (SDH).

4 $$\:Precision=\frac{TP}{TP+FP}\times\:100$$

### F1-score

The harmonic mean of precision and sensitivity (recall). This metric provides a balance between precision and recall, especially when the class distribution is imbalanced.

5 $$\:F1-score=\frac{2.TP}{2.TP+FP+FN}\times\:100$$

### Intersection over union (IoU)

Although IoU is conventionally a segmentation metric, in this study, it was adapted for classification using confusion matrix values.

6 $$\:IoU=\frac{TP}{TP+FP+FN}\times\:100$$

### Dice similarity coefficient (DSC)

DSC was adapted for classification using confusion matrix values instead of pixel-wise masks.

7 $$\:DSC=\frac{2.TP}{(TP+FP)+(TP+FN)}\times\:100$$

### AUC-ROC

This metric evaluates the model’s ability to distinguish each target class from all other classes. For each class in the one-vs-rest (OvR) scheme, it measures the area under the curve plotted between the TP Rate (Sensitivity) and the FP Rate (1 − Specificity). ROC curves and AUC values were computed for each class in every k-fold, and overall performance was reported using macro-averaged AUC across classes. A higher AUC indicates better model performance.

### Limitations and future directions

Despite the encouraging results demonstrated in this study, several limitations must be acknowledged, and these also inform directions for future research. Although the classification of SDH stages was performed under the direct supervision of a board-certified radiologist, there remains a possibility that the model predominantly learned from numerical patterns, particularly HU intensities, rather than fully capturing the nuanced diagnostic reasoning employed by clinicians. While HU values were retrospectively measured to describe typical characteristics within each SDH stage, they were not used as strict labeling criteria. To enhance clinical validity, future work will incorporate blinded multi-radiologist consensus labeling and inter-rater agreement analysis to more accurately reflect expert diagnostic interpretations.

The present study did not include a comparison with a rule-based HU threshold classifier. Although such methods are limited in clinical practice due to HU value overlaps, scan variability, and the need for manual region-of-interest selection, implementing a baseline HU-based model in future work would allow for a clearer quantification of the diagnostic benefit added by the CNN-based approach. Furthermore, clinically ambiguous cases, such as mixed-density hematomas, low-quality scans, or overlapping HU presentations were deliberately excluded during dataset curation to maintain ground-truth consistency. Recognizing their importance in real-world clinical scenarios, future investigations will include these diagnostically challenging cases to better evaluate the model’s robustness and practical utility.

This study also did not involve a formal comparison with multiple human radiologists, nor did it assess combined AI-human diagnostic performance. While agreement with a single expert was quantified using Cohen’s kappa coefficient, future observer studies are planned to evaluate whether AI assistance improves diagnostic accuracy or reduces interpretive ambiguity, especially in borderline cases. Another limitation lies in the dataset’s de-identified nature and modest sample size. Although the RSNA dataset draws from multiple institutions and diverse imaging protocols, the absence of patient-specific metadata (e.g., age, sex, medical history) limits the ability to evaluate potential biases or confounding factors. Expanding the dataset with broader demographic representation and clinically enriched annotations is a planned next step.

From a technical perspective, comparisons with state-of-the-art deep learning architectures such as ResNet or DenseNet were not performed due to computational constraints. Future studies will incorporate such benchmarking to evaluate relative performance under standardized conditions. Although overfitting was mitigated through dropout, data augmentation, and cross-validation, additional strategies such as early stopping or L2 regularization could further enhance generalizability. Lastly, while visualizations such as confusion matrices and ROC curves were included, future work will expand interpretability efforts by integrating Grad-CAM heatmaps and representative prediction overlays. These additions will support clearer insight into model decision-making and strengthen its potential integration into clinical workflows.

### Ethics/IRB statement

This retrospective study used only publicly available, fully de-identified CT images from the RSNA Intracranial Hemorrhage Detection dataset (Kaggle). The Ethics Review Committee, Faculty of Allied Health Sciences, University of Peradeniya, Sri Lanka, reviewed the study protocol, determined that it was exempt from formal ethical approval, and waived the requirement for informed consent.

## Supplementary Information

Below is the link to the electronic supplementary material.

Supplementary Material 1

## Data Availability

All data analyzed in this study were from the publicly available RSNA Intracranial Hemorrhage Detection dataset hosted on Kaggle. Access requires free registration on Kaggle and agreement with the competition terms; the authors had no special access privileges. The dataset page is available at: https://www.kaggle.com/competitions/rsna-intracranial-hemorrhage-detection/data.

## Abbreviations

CT: Computed tomography
NCCT: Non-contrast computed tomography
ICH: Intracranial hemorrhage
SDH: Subdural hemorrhage
CSF: Cerebrospinal fluid
HTN: Hypertension
RSNA: Radiological Society of North America
DICOM: Digital Imaging and Communications in Medicine
GPU: Graphics processing unit
TPU: Tensor processing unit
CNN: Convolutional neural network
FCN: Fully convolutional network
AUC-ROC: Area under the receiver operating characteristic curve
DSC: Dice similarity coefficient
IoU: Intersection over Union
