# Supplementary material for: A deep learning approach to predict temporal changes of subdural hemorrhage on computed tomography
Source: Sci Rep. 2025 Oct 29;15:37826. doi: 10.1038/s41598-025-21721-z (PMC12572109; doi:10.1038/s41598-025-21721-z)
Supplement: Supplementary file 1 — Supplementary Material 1 [file 41598_2025_21721_MOESM1_ESM.pdf]

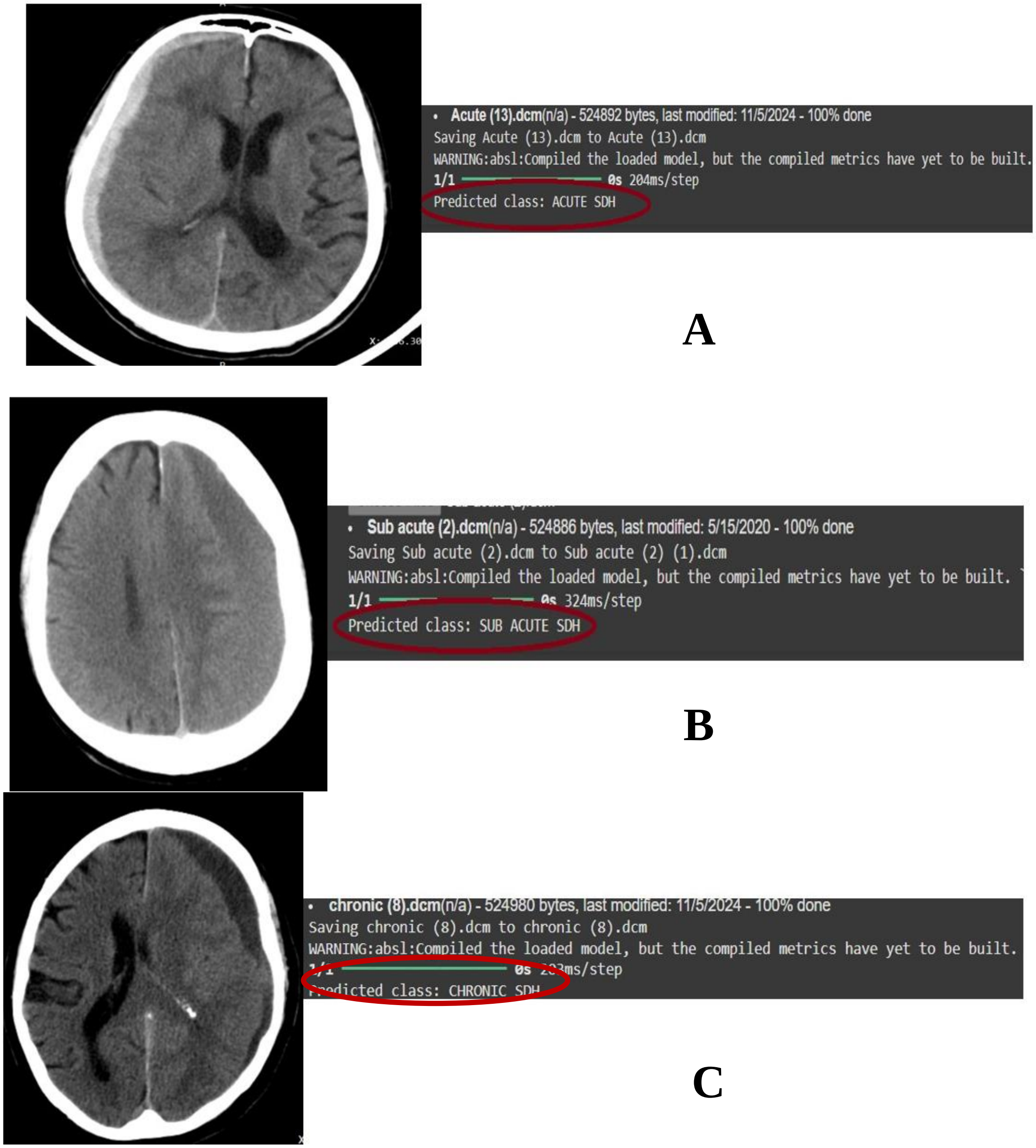
Acute (13).dcm(n/a) - 524892 bytes, last modified: 11/5/2024 - 100% done
Saving Acute (13).dcm to Acute (13).dcm
WARNING:absl:Compiled the loaded model, but the compiled metrics have yet to be built.
1/1 0s 204ms/step
Predicted class: ACUTE SDH
A
Sub acute (2).dcm(n/a) - 524886 bytes, last modified: 5/15/2020 - 100% done
Saving Sub acute (2).dcm to Sub acute (2) (1).dcm
WARNING:absl:Compiled the loaded model, but the compiled metrics have yet to be built.
1/1 0s 324ms/step
Predicted class: SUB ACUTE SDH
B
chronic (8).dcm(n/a) - 524980 bytes, last modified: 11/5/2024 - 100% done
Saving chronic (8).dcm to chronic (8).dcm
WARNING:absl:Compiled the loaded model, but the compiled metrics have yet to be built.
CHRONIC SDH
C

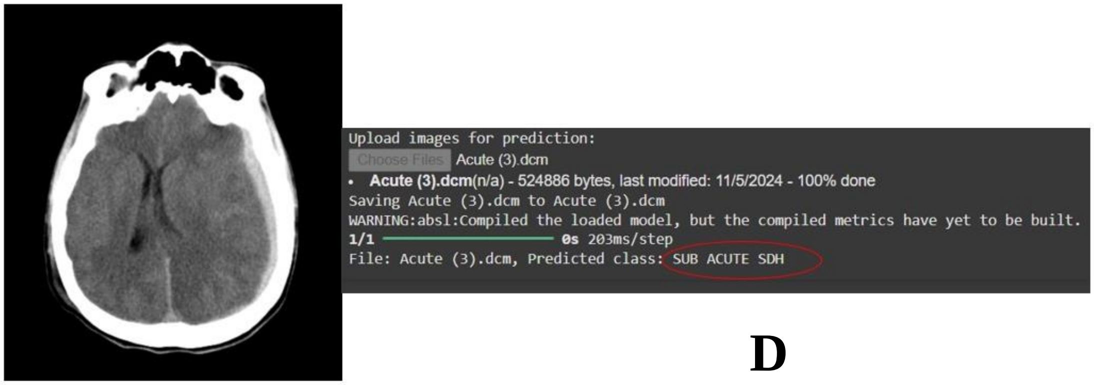

D

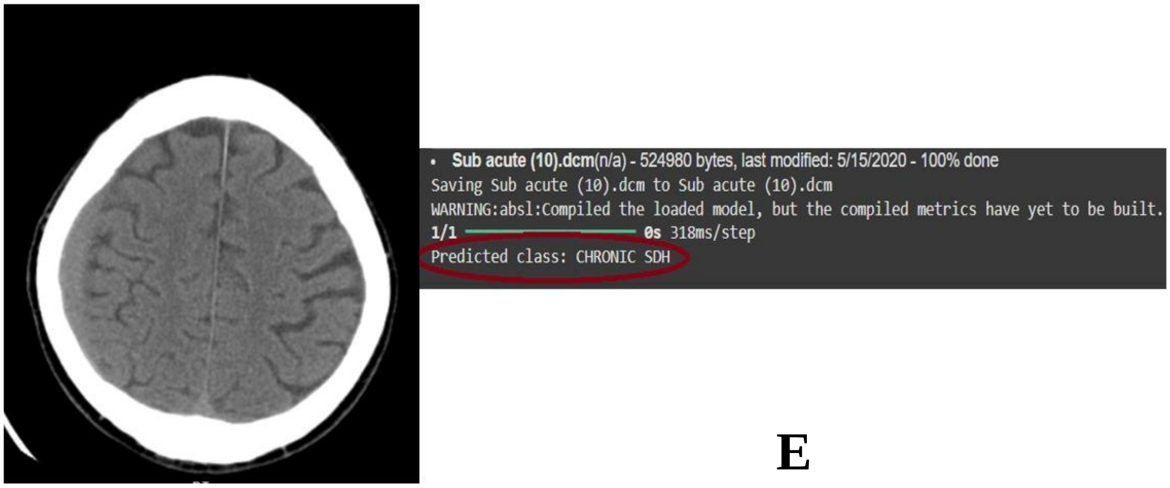

E

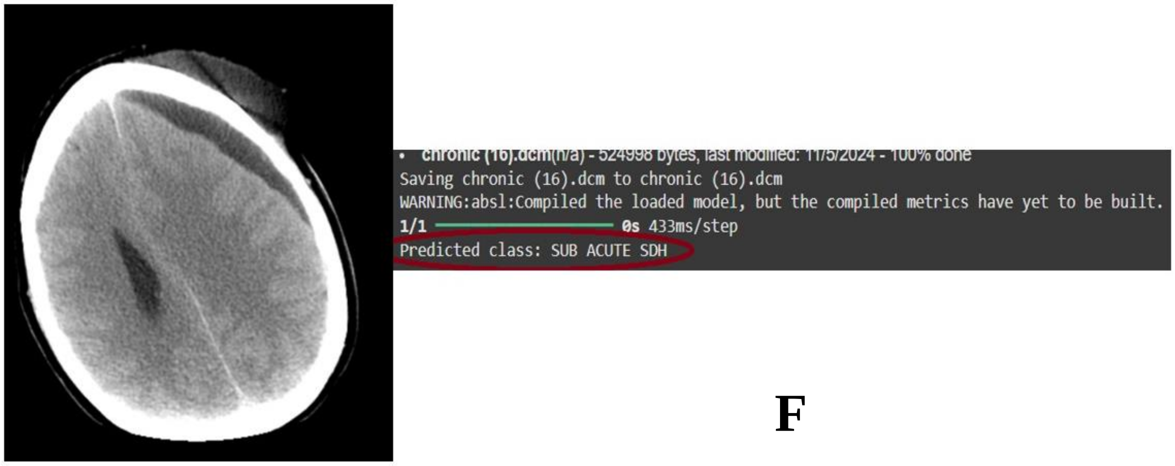

F

Figure S1. Representative CT slices illustrating the model’s classification of SDH stages. The images include correctly predicted cases of Acute, Subacute, and Chronic SDH ((A),(B)and(C)), as well as examples of misclassifications such as Acute predicted as Subacute (D), Subacute predicted as Chronic (E), and Chronic predicted as Subacute (F).

Metric	Acute SDH	Subacute SDH	Chronic SDH
DSC	87.25 %	80.77 %	88.28 %
IoU	77.38 %	67.74 %	79.01 %

Table S1. Case-level Dice Similarity Coefficient (DSC) and Intersection over Union (IoU) values for Acute, Subacute, and Chronic SDH classifications. These exploratory overlap metrics were adapted from confusion matrices to provide an additional perspective on classification performance.
